# Serological and Molecular Characterization of *Mycobacterium avium* Subsp. *paratuberculosis* (MAP) from Sheep, Goats, Cattle and Camels in the Eastern Province, Saudi Arabia

**DOI:** 10.3390/ani11020323

**Published:** 2021-01-28

**Authors:** Ibrahim Elsohaby, Mahmoud Fayez, Mohamed Alkafafy, Mohamed Refaat, Theeb Al-Marri, Fanan A. Alaql, Abdulaziz S. Al Amer, Abdelmonem Abdallah, Ahmed Elmoslemany

**Affiliations:** 1 Department of Animal Medicine, Faculty of Veterinary Medicine, Zagazig University, Zagazig 44511, Egypt; abd.el.monem.ali@umontreal.ca; 2 Department of Health Management, Atlantic Veterinary College, University of Prince Edward Island, Charlottetown, PEI C1A 4P3, Canada; 3 Department of Bacteriology, Veterinary Serum and Vaccine Research Institute, Ministry of Agriculture, Cairo 131, Egypt; mahmoudfayez30@hotmail.com; 4 Al Ahsa Veterinary Diagnostic Lab, Ministry of Environment, Water and Agriculture, Al-Ahsa 31982, Saudi Arabia; theep8@hotmail.com (T.A.-M.); dr-aziz18@hotmail.com (A.S.A.A.); 5 Department of Biotechnology, College of Science, Taif University, P.O. Box 11099, Taif 21944, Saudi Arabia; m.kafafy@tu.edu.sa; 6 Department of Pathology, Animal Health Research Institute, Dokki, Giza 12618, Egypt; refaatpath@yahoo.com; 7 Department of Microbiology, College of Science, King Saud University, Riyadh 11451, Saudi Arabia; fanan.abdulaziz@gmail.com; 8 Hygiene and Preventive Medicine Department, Faculty of Veterinary Medicine, Kafrelsheikh University, Kafr El-Sheikh 33516, Egypt; aelmoslemany@gmail.com

**Keywords:** MAP, ELISA, PCR, IS*900* gene, ruminants, Saudi Arabia

## Abstract

**Simple Summary:**

*Mycobacterium avium* subsp. *paratuberculosis* (MAP) is the causative agent of Johne’s disease, affecting small and large ruminants and causing chronic diarrhea and severe emaciation. MAP is prevalent in many countries, including Saudi Arabia. Serological and molecular characterization of MAP and determination of the prevalent strains are essential for the control strategies. The results obtained from 31 herds showed that the sheep type (S-type) was the most prevalent MAP type and the molecular characterization revealed different strain profiles distributed among the sheep, goat, cattle, and camel herds in Eastern Province, Saudi Arabia.

**Abstract:**

The objectives of the present study were to characterize *Mycobacterium avium* subsp. *paratuberculosis* (MAP) infection using serological and molecular tools and investigate the distribution and molecular characterization of MAP strains (cattle (C) and sheep (S) types) in sheep, goat, cattle, and camel herds in Eastern Province, Saudi Arabia. Serum and fecal samples were collected from all animals aged >2 years old in 31 herds (sheep = 8, goats = 6, cattle = 8 and camels = 9) from January to December 2019. Serum samples were tested by ELISA for the detection of MAP antibodies. Fecal samples were tested by PCR for the detection of MAP IS*900* gene and the identification of MAP strains. MAP antibodies were detected in 19 (61.3%) herds. At the animal level, antibodies against MAP were detected in 43 (19.5%) sheep, 21 (17.1%) goats, 13 (19.7%) cattle and 22 (9.1%) camels. The IS*900* gene of MAP was detected in 23 (74.2%) herds and was directly amplified from fecal samples of 59 (26.8%) sheep, 34 (27.6%) goats, 20 (30.3%) cattle and 36 (15.0%) camels. The S-type was the most prevalent MAP type identified in 15 herds, and all were identified as type-I, while the C-type was identified in only 8 herds. The IS*900* sequences revealed genetic differences among the MAP isolates recovered from sheep, goats, cattle and camels. Results from the present study show that MAP was prevalent and confirm the distribution of different MAP strains in sheep, goat, cattle and camel herds in Eastern Province, Saudi Arabia.

## 1. Introduction

*Mycobacterium avium* subsp. *paratuberculosis* (MAP) is a slow-growing, Gram-positive acid-fast bacterium that infects domestic and wild ruminants causing paratuberculosis or Johne’s disease [1,2]. Paratuberculosis is a chronic wasting disease that infects small and large ruminants causing chronic diarrhea, severe weight loss, emaciation and reduction in milk and wool production [3]. Infected animals have the ability to shed MAP in feces and milk for up to two years before the onset of clinical signs [4], which could transmit MAP to susceptible animals via fecal–oral route [5]. In humans, the MAP was related to Crohn’s disease, but this relationship remains controversial [6,7].

To reduce the risk of MAP infection, infected animals should be detected and culled at an early stage of infection [8]. There are currently several methods available for direct (i.e., bacterial culture and PCR) and indirect (i.e., ELISA and interferon-γ) detection of MAP infection. Bacterial culture is expensive, slow, takes up to 42 days or longer and not available in all laboratories [9]. However, PCR is more rapid and frequently used to remove high shedders from herds [10]. In addition, PCR could be used to directly diagnose MAP from fecal samples with sensitivity between 70% and 100% [11,12] and specificity of 100% [12,13]. The majority of previous studies that used PCR to detect MAP have targeted the IS*900* gene, which represents approximately 17 copies of the MAP genome, thus providing a higher level of sensitivity [14].

Historically, MAP has been classified into cattle (C) and sheep (S) types based on the host from which MAP was first isolated [15]. Recently, phenotypic characteristics and molecular biology classified MAP into type-I, type-II and type-III [16,17,18]. MAP type-I was a slow grower, takes more than 16 weeks to achieve visible growth and was strongly associated with sheep. However, MAP type-II takes 4 to 16 weeks to grow and is commonly isolated from cattle [19]. MAP type-III or called “intermediate” was primarily thought to be different from C-type and S-type [19], but a recent study based on whole-genome sequence revealed that both types (I and III) are subgroups of S-type [20].

MAP infection has been reported in sheep [21], goats [22], cattle [23] and camels [24,25] in Saudi Arabia. However, to the author’s knowledge, there is limited information about the molecular characterization of MAP and the MAP types distributed in sheep, goat, cattle and camel herds in Saudi Arabia. Thus, the objectives of the present study were to (1) determine the MAP infection in sheep, goat, cattle and camel herds using serological and molecular tools and (2) investigate the distribution and molecular characterization of MAP strains (C-type and S-type) in sheep, goat, cattle and camel herds in Eastern Province, Saudi Arabia.

## 2. Materials and Methods

### 2.1. Study Population

**Figure 1:**
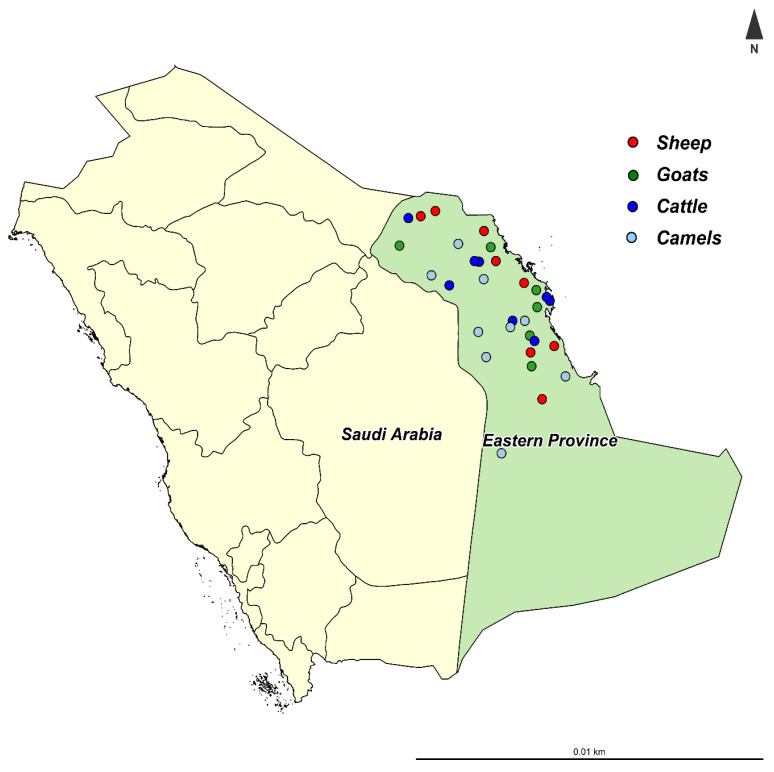
The location of Eastern Province in Saudi Arabia and the location of sampled herds.

The present study was performed in the Eastern Province of Saudi Arabia, which is located at 22°30′ N, 51°00′ E, at 390 km from the capital Riyadh (Figure 1). The Eastern Province is the third most populous province in Saudi Arabia, with varying climatic conditions from semi-desert to desert. The Eastern Province shares the borders with five countries (Iraq, Kuwait, Oman, Qatar, and the United Arab Emirates), which may increase the risk of pathogen introduction to the country. 

### 2.2. Samples Collection 

A cross-sectional study was carried out from January to December 2019 to select herds with a previous history of Johne’s disease. A total of 31 herds were investigated (sheep = 8, goats = 6, cattle = 8 and camels = 9). Within herds, blood and rectal scrapings mixed with feces were collected from each animal aged >2 years old (Table 1). Blood samples were obtained from jugular venipuncture using vacuum tubes without anticoagulants; however, fecal samples were collected directly from the rectum into plastic containers. Samples were labeled with herd and animal ID, species and date of collection, then sent to the laboratory in ice pack containers and processed within 24 h.

According to the local and national regulations, the Ethics Committee of Taif University has approved the study protocol (TURSP-2020-57). An informed consent (written in Arabic) was signed by all herd owners approving the use of their specimen samples for research purposes before enrolment in this study.

### 2.3. Serological Identification of MAP in Serum Samples

A commercial ELISA kit (mycobacterium paratuberculosis antibody test kit) from IDEXX (IDEXX, Hoofddorp, Netherlands) was used to detect antibodies against MAP in serum samples. Samples and controls were diluted 1:20 in dilutant containing mycobacterium phlei extract, then incubated at room temperature for 1 h to neutralize the cross-reactions with atypical mycobacteria. Briefly, a 100 µL/well of the diluted sample was loaded into the 96-well microtiter plate and then incubated at room temp for 45 mins. The conjugated and Tetramethylbenzidine (TMB) substate were added according to the manufacturer’s instructions. The addition of the stop solution stopped color development, and the optical density (OD) in each well was measured at 450 nm. Results were expressed as the ratio (S/P ratio) of the sample OD minus the mean of the negative control OD to the mean positive control OD minus the mean negative control OD. The sample was considered positive if the S/P ratio was ≥55%. 

### 2.4. Molecular Identification of MAP in Fecal Samples

The DNA was extracted from each fecal sample according to Clark et al. [11] method with some modifications. Briefly, two grams of fecal sample were mixed with 4 mL sterile distilled water and vortexed vigorously for 15 s, then kept stand at room temperature for 10 min. Using a sterile pipette, 1.5 mL from the supernatant was transferred to a 2 mL tube containing 1 g zirconia/silica beads and centrifuged for 10 min at 13,000× *g*. After discarding the supernatant, the pellet was suspended in 400 µL distilled water and incubated at 95 °C for 10 min, then disrupted for 45 s at 6500 rpm using MagNA Lyser (Roche Diagnostics, Basel, Switzerland). The sample was then centrifuged for 10 min at 13,000× *g*. The DNA was purified from the supernatant using QIAamp^®^ PowerFecal^®^ kit (QIAGEN, Courtaboeuf, France) according to the manufacturer’s instructions and the purified DNA was stored at froze temperature.

The template DNA was subjected to three different PCRs for molecular detection of MAP in fecal samples and identifying both cattle and sheep types. The purified DNA was amplified to detect the insertion sequence IS*900* of MAP as described previously [17]. A specific PCR (DMC-PCR) to differentiate between cattle (C-type) and sheep (S-type) strains was performed according to Collins, et al. [26]. The size of the expected amplicon would be 310 bp for C-type and 162 bp for S-type. The representational difference analysis (RDA) fragments (pig-RDA10, pig-RDA20, and pig-RDA30) specific for sheep MAP type-I was amplified [27]. HotStartTaq^®^ plus master mix kit (QIAGEN, Germantown, MD, USA) was used to amplify 2 µL of each purified genomic DNA, and PCR conditions were performed using I cycler PCR machine (BIO-RAD, Hercules, CA, USA). The primers, annealing temperature, and expected DNA product size for each PCR conditions are given in Table 2.

The amplified IS*900* product was purified (QIAquick PCR Purification Kit, QIAGEN GmbH—Hilden, Germany) and subsequently sequenced (3500 Genetic Analyzer, Applied Biosystems, Foster City, CA, USA). The IS*900* sequences were aligned with the IS*900* elements of the reference MAP K-10 reference genome (GenBank accession no. AE016958). 

### 2.5. Data Analysis 

Data were introduced into R version 3.5.3 for visualization and descriptive data analysis. The proportions of MAP infections in each herd were calculated from the ratio of MAP positive samples to the total number of tested samples, with the exact binomial confidence interval of 95% (95% CI).

## 3. Results

### 3.1. Clinical and Post-Mortem Lesions

**Figure 2:**
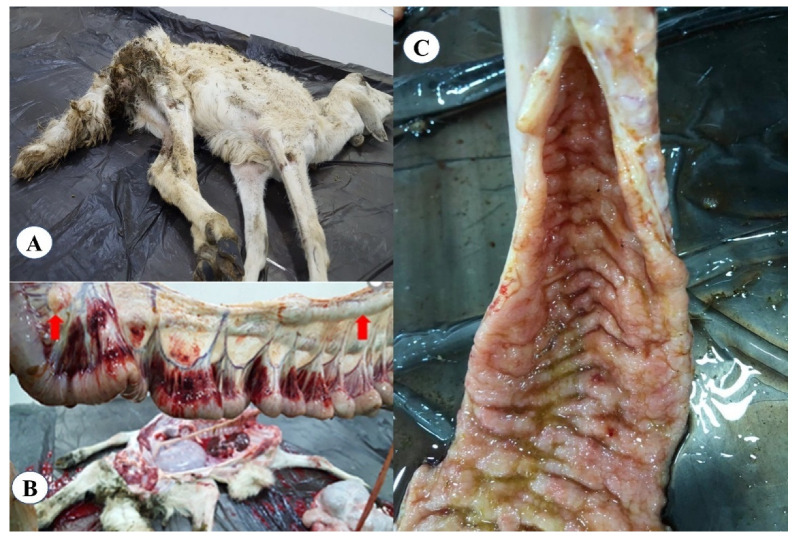
Clinical and gross lesions of *Mycobacterium avium* subsp. *paratuberculosis* infected goat. (**A**) dead goat with signs of diarrhea and emaciation; (**B**) mesenteric lymph nodes were enlarged and edematous; and (**C**) thickened and corrugated ileum.

From the 31 herds included in the study, only 7 (sheep = 2, goat = 2, cattle = 1 and camel = 2) herds showed one or more clinical signs of Johne’s disease, including chronic diarrhea and severe weight losses (Figure 2A). Necropsy findings in a single representative dead goat showed enlarged and edematous mesenteric lymph nodes (Figure 2B) and thickened and corrugated ileum (Figure 2C).

### 3.2. Serological Detection of MAP

At the herd level, MAP antibodies were detected in 19 (61.3%) herds, including 6 sheep, 5 goat, 4 cattle and 4 camel herds. However, at the animal level, antibodies against MAP were detected in 43 (19.5%) sheep, 21 (17.1%) goats, 13 (19.7%) cattle and 22 (9.1%) camels (Table 3).

### 3.3. Molecular Detection of MAP

**Figure 3:**
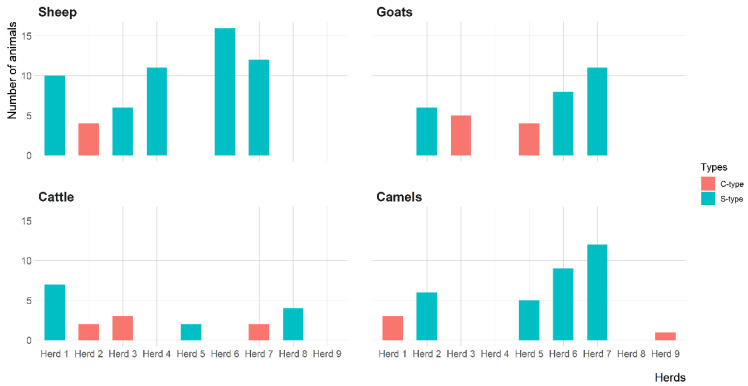
The distribution of *Mycobacterium avium* subsp. *paratuberculosis* strains (C-type and S-type) in 31 herds with a previous MAP infection history in Eastern Province, Saudi Arabia.

The IS*900* gene of MAP was detected in 23 (74.2%) herds, including 6 sheep, 5 goat, 6 cattle and 6 camel herds. However, the IS*900* gene of MAP was directly amplified from fecal samples of 59 (26.8%) sheep, 34 (27.6%) goats, 20 (30.3%) cattle and 36 (15.0%) camels (Table 3). Figure 3 shows the distribution of MAP S-type and C-type in sheep, goat, cattle and camel herds. The S-type was the most prevalent MAP type identified in 15 herds, including 5 sheep, 3 goats, 3 cattle and 4 camel herds. All S-types were identified as type-I based on RDA fragments PCR. The C-type was identified in 8 herds, including 1 sheep, 2 goats, 3 cattle and 2 camel herds.

**Figure 4:**
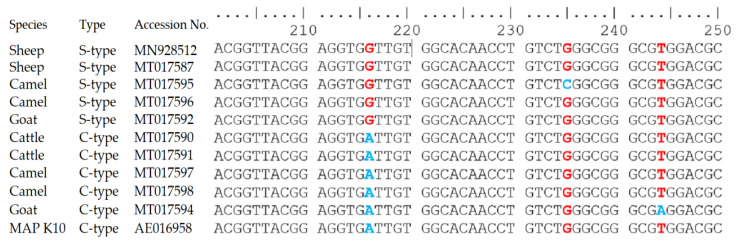
The difference in the sequences of the IS*900* gene of *Mycobacterium avium* subsp. *paratuberculosis* strains (C-type and S-type) recovered from sheep, goat, cattle and camel herds. The single nucleotide polymorphism (SNP) in red and blue colours represent the differences between S-type and C-type at position 216, 235 and 244.

The differences in the IS*900* gene sequences of MAP S-type and C-type identified in sheep, goat, cattle and camel are presented in Figure 4. All S-type sequences from sheep, cattle, goats and camels showed single nucleotide polymorphism (SNP) (G instead of A) at position 216 (GenBank accession no: MN928512, MT017587, MT017592 and MT017596). Additional SNP (C instead of G) was detected at position 235 in one S-type sequence from the camel (GenBank accession no: MT017595). However, the sequences of IS*900* gene of C-type from sheep, goat, cattle and camel (GenBank accession no: MT017590, MT017591, MT017594 and MT017598) showed 100% identity to IS*900* elements of MAP K10 strain. A single nucleotide polymorphism (SNP) (A instead of T) was detected at position 244 in C-type IS*900* sequence from the camel (GenBank accession no: MT017594).

## 4. Discussion

Johne’s disease is a chronic contagious enteric disease with significant economic losses, particularly in the dairy industry around the world [28]. Early detection of MAP infection is essential for the successful control of Johne’s disease. Clinically, animals with Johne’s disease are classified under four categories based on the severity of clinical signs, the shedding of causative agents and the efficiency of diagnosis using existing laboratory techniques [29]. Animals up to 2 years of age are often in the early stage (silent infection) where no clinical signs and no cost-efficient screening tools can identify infection [30]. Bacterial culture of MAP from feces and tissues has been accepted as the reference standard for MAP infection diagnosis [31,32,33]. However, the long incubation time, the high expense and the need for a professional mycobacteriology laboratory are common disadvantages. Therefore, ELISA and PCR were commonly used as it rapid, simple, sensitive and cost-efficient tools for diagnosing MAP infection [34]. However, ELISA sensitivity is limited by the immune response to MAP infection, where antibodies can be detected at the late stage of infection and after fecal shedding has begun.

In the present study, herds with a previous history of MAP infection were selected, and animals aged over 2 years were sampled to increase the likelihood of serological and molecular detection of MAP [30,35]. The MAP antibodies were detected in sheep, goats, cattle and camels. However, the highest ELISA positive rate was in sheep (19.5%) and cattle (19.7%). Although the prevalence determination in the present study was biased due to the analysis of herds with previous MAP infection history, the calculated animal level prevalences were lower than those reported in European countries [36,37,38]. The proportion of ELISA positive sheep and goats was higher than the 6.3% reported in Italy [36] and lower than the 48.3% reported in Canada [39]. However, the proportion of ELISA positive detected in cattle was higher than the 0.4% recorded in Slovenia [37], lower than the 24.1% recorded in Germany [38] and similar to the 19% recorded in cattle in Australia [40]. In camel, 9.1% of the current study samples were ELISA positive, which was lower than the 30% previously reported in Saudi Arabia [25]. The variations in ELISA results in the diagnosis of MAP antibodies may be attributed to the delay of the humoral immune response of infected animals and the variability of antigens in commercial ELISA kits used in each country [30,31].

Animals in the present study were also tested by PCR for the detection of MAP IS*900* gene. Several studies detected MAP infected animals through the detection of IS*900* gene [11,41,42], which is highly specific and can detect 10-100 MAP per gram of feces or milk [43,44]. In the present study, the frequency of MAP infected animals detected by PCR was higher than ELISA. These results were similar to that reported by Clark et al. [11] and contradicted results reported by Wells et al. [45], who found that ELISA outperformed PCR in light and moderate shedding animals. The obtained result was not surprising because MAP-infected animals produce an immune response at the late stage of infection [39,46], whereas infected animals have the ability to shed the bacterium in feces and milk in an early stage of infection and about two years before the onset of clinical signs [4].

The phenotypic and genotypic differentiation of MAP provides essential information for molecular epidemiological analysis [47]. Several DNA-based techniques have been developed to examine the transmission of MAP among different animal species [16,48]. In the study area, S-type was the predominated MAP strain; it was identified by DMC-PCR in 15 herds, whereas the C-type was identified only in 8 herds. DMC-PCR was previously used to differentiate C-type from S-type [15]. The C-type is usually isolated from cattle; however, it shows no host specificity and can be isolated from a wide range of animals, including non-ruminants [49,50]. In the past, it was believed that S-type has a preference for sheep and goats. However, recently it has been isolated from cattle, deer and camelids [51]. A single nucleotide polymorphism was detected at position 216 after comparing the IS*900* sequences of S-type with its ancients from MAP K10 strain, whereas the C-type showed 100% homology. Similar results were observed previously elsewhere [17,52,53,54].

It should be noted that our study has some limitations regarding the mycobacterial culture. We agree that fecal culture is the reference method for the detection of MAP. However, culture is expensive, slow and not widely available in Saudi Arabia. The direct fecal PCR used in the present study was sensitive (sensitivity ranged from 70% to 100% based on the MAP infection stage) and specific [11,12,13].

## 5. Conclusions

To the best of our knowledge, this is the first study reporting the molecular characterization of MAP recovered from small and large ruminants in Saudi Arabia. The present study results showed that MAP was prevalent and confirm the distribution of different MAP strains in sheep, goat, cattle, and camel herds in Eastern Province, Saudi Arabia.

## Figures and Tables

**Table 1:** The total number (*n*) of animals in each herd and number of animals (>2 years-old) sampled from each herd with a previous history of *Mycobacterium avium* subsp. *paratuberculosis* infection in Eastern Province, Saudi Arabia.

Herds	Sheep		Goats		Cattle		Camels	
	*n*	>2 Years	*n*	>2 Years	*n*	>2 Years	*n*	>2 Years
1	50	28	60	38	20	11	40	22
2	20	11	50	21	15	9	50	31
3	35	19	30	19	10	8	40	35
4	60	32	20	11	8	8	15	10
5	15	6	25	11	19	10	49	27
6	90	55	45	23	11	7	68	41
7	85	47	-	-	9	6	73	39
8	40	22	-	-	13	7	30	19
9	-	-	-	-	-	-	26	16
Total		220		123		66		240

**Table 2:** Primers, annealing temperatures and product size used for *Mycobacterium avium* subsp. *paratuberculosis* identification in the present study.

Primer Name	Primer Sequences	Annealing (°C)	Product Size (bp)	References
IS*900*-F	5′ CCTTTCTTGAAGGGTGTTCG 3′	58	548	[17]
IS*900*-R	5′ CCACCAGATCGGAACGTC 3′			
DMC1-529 F	5′ GCTGTTGGCTGCGTCATGAAG 3′	60	310 (C-type)	[26]
DMC1-531 F	5′ TCTTATCGGACTTCTTCT GGC 3′		162 (S-type)	
DMC1-533 R	5′ CGGATTGACCTGCGTTTCAC 3′			
pig-RDA10 P19	5′ TAG CGG TCC CGC AGT TTG GC 3′	61	382	[27]
pig-RDA10 P20	5′ TCA AGC CGA ACG AGG TGG TCG 3′			
pig-RDA20 P21	5′ TCG TCC CGT CCC GAT GCT GT 3′		560	
pig-RDA20 P22	5′ TGA GTC CTG TCG TGC ATG CG 3′			
pig-RDA30 P23	5′ TGA AGA GCC CGG ACA AGG GG 3′		525	
pig-RDA30 P24	5′ TAG GTC TCA GTG GTC CAC CAG C 3′			

**Table 3:** Number and percentage of ELISA and PCR positive animals in 31 herds examined for *Mycobacterium avium* subsp. *paratuberculosis* in Eastern Province, Saudi Arabia.

Herds	Sheep			Goats			Cattle			Camels		
	No. of Samples	No. of ELISA Positive (%)	No. of IS*900* Positive (%)	No. of Samples	No. of ELISA Positive (%)	No. of IS*900* Positive (%)	No. of Samples	No. of ELISA Positive (%)	No. of IS*900* Positive (%)	No. of Samples	No. of ELISA Positive (%)	No. of IS*900* Positive (%)
1	28	6 (21.4)	10 (35.7)	38	6 (15.8)	11 (28.9)	11	6 (54.5)	7 (63.6)	22	0 (0.0)	2 (9.1)
2	11	2 (18.2)	4 (36.4)	21	3 (14.3)	6 (28.6)	9	0 (0.0)	2 (22.2)	31	3 (9.7)	5 (16.1)
3	19	5 (26.3)	8 (42.1)	19	7 (36.8)	5 (26.3)	8	2 (25.0)	3 (37.5)	35	0 (0.0)	0 (0.0)
4	32	7 (21.9)	9 (28.1)	11	0 (0.0)	0 (0.0)	8	0 (0.0)	0 (0.0)	10	0 (0.0)	0 (0.0)
5	6	0 (0.0)	0 (0.0)	11	2 (18.2)	4 (36.4)	10	0 (0.0)	2 (20.0)	27	3 (11.1)	2 (7.4)
6	55	12 (21.8)	16 (29.1)	23	3 (13.0)	8 (34.8)	7	0 (0.0)	0 (0.0)	41	6 (14.6)	8 (19.5)
7	47	11 (23.4)	12 (25.5)	-	-	-	6	2 (33.3)	2 (33.3)	39	10 (25.6)	6 (15.4)
8	22	0 (0.0)	0 (0.0)	-	-	-	7	3 (42.9)	4 (57.1)	19	0 (0.0)	0 (0.0)
9	-	-	-	-	-	-	-	-	-	16	0 (0.0)	1 (6.3)
Total	220	43 (19.5)	59 (26.8)	123	21 (17.1)	34 (27.6)	66	13 (19.7)	20 (30.3)	240	22 (9.1)	24 (10.0)

## Data Availability

The data presented in this study are available on request from the corresponding author.
